# Detecting the drip: pediatric neuroradiologist guide to spinal CSF leaks

**DOI:** 10.1007/s00247-026-06764-2

**Published:** 2026-09-02

**Authors:** Mark D. Mamlouk

**Affiliations:** 1 Department of Radiology, Kaiser Permanente Santa Clara Medical Center, 700 Lawrence Expressway, Department 104, Santa Clara, CA 95051 USA; 2 Department of Radiology and Biomedical Imaging, University of California, San Francisco, San Francisco, CA USA

**Keywords:** Pediatric spinal CSF leaks, Spontaneous intracranial hypotension, CSF-venous fistula, Epidural blood patch

## Abstract

Pediatric spinal CSF leaks are uncommon and encompass a spectrum of leak types with distinct imaging findings, diagnostic pathways, and treatment strategies. Brain and spine MRI guide the evaluation of suspected CSF leaks and the selection of advanced imaging techniques for precise leak localization. Leak types include dural tears, CSF-venous fistulas, and CSF-vascular malformation fistulas, each with distinct imaging and treatment considerations. This pictorial review illustrates the spectrum of pediatric spinal CSF leaks and provides a practical framework for their diagnosis and management.

## Introduction

Pediatric spinal CSF leaks are rare and are frequently underrecognized, often resulting in delayed diagnosis and prolonged symptoms. They may occur following trauma or medical procedures or arise spontaneously from a variety of spinal dural defects and fistulous communications. Similar to adults, affected children often present with various symptoms and may undergo evaluation by multiple specialists before the diagnosis is established. Because MRI and myelography are often essential to the detection and localization of spinal CSF leaks, the neuroradiologist plays a critical role in both the diagnosis and the image-guided treatment [1]. This pictorial review provides a practical overview of the clinical presentation, classification, imaging algorithms, and contemporary management of pediatric spinal CSF leaks.

## Clinical symptoms and presentation

Spinal CSF leaks result in a loss of CSF, which can lead to intracranial hypotension. The hallmark feature of intracranial hypotension is orthostatic headache, which is typically worse when upright and relieved while supine. Valsalva maneuvers, such as coughing, sneezing, and bending over, can worsen headache symptoms [2]. One major challenge in diagnosis is that not all patients present with orthostatic headaches, and headaches can wane with time, potentially causing providers to overlook this diagnosis [3]. In addition to headaches, patients can present with neck pain, muffled hearing, tinnitus, vertigo, and cranial neuropathies.

## Spinal CSF leak types and etiologies

Spinal CSF leaks can be broadly categorized as nonspontaneous (iatrogenic or traumatic) and spontaneous causes (Table 1). Because the imaging and treatment algorithms may differ between these causes, a detailed clinical history is pertinent to direct the appropriate pathway.

**Table 1:** Classification, imaging findings, and treatment of pediatric CSF leak types

Leak type	Spine MRI	Preferred myelography	Treatment
Ventral dural tear	SLEC positive	Prone CTM or DSM	Targeted blood or fibrin glue patch; Surgery
Lateral dural tear	SLEC positive	Decubitus CTM or DSM	Targeted blood or fibrin glue patch; Surgery
CVF	-SLEC negative -Meningeal diverticula	Decubitus CTM or DSM	Targeted fibrin glue patch; Embolization; Surgery
CSF-vascular malformation fistula	-SLEC negative -Paraspinal lesion	-Decubitus CTM or DSM	Targeted fibrin glue patch; Embolization; Surgery
Arachnoid bleb	-SLEC negative -Focal T2 signal at bleb	Supine CTM	Surgery

*SLEC*, spinal longitudinal extradural collection; *CVF*, CSF-venous fistula; *CTM*, CT myelography; *DSM*, digital subtraction myelography

### Nonspontaneous CSF leaks

**Fig. 1:**
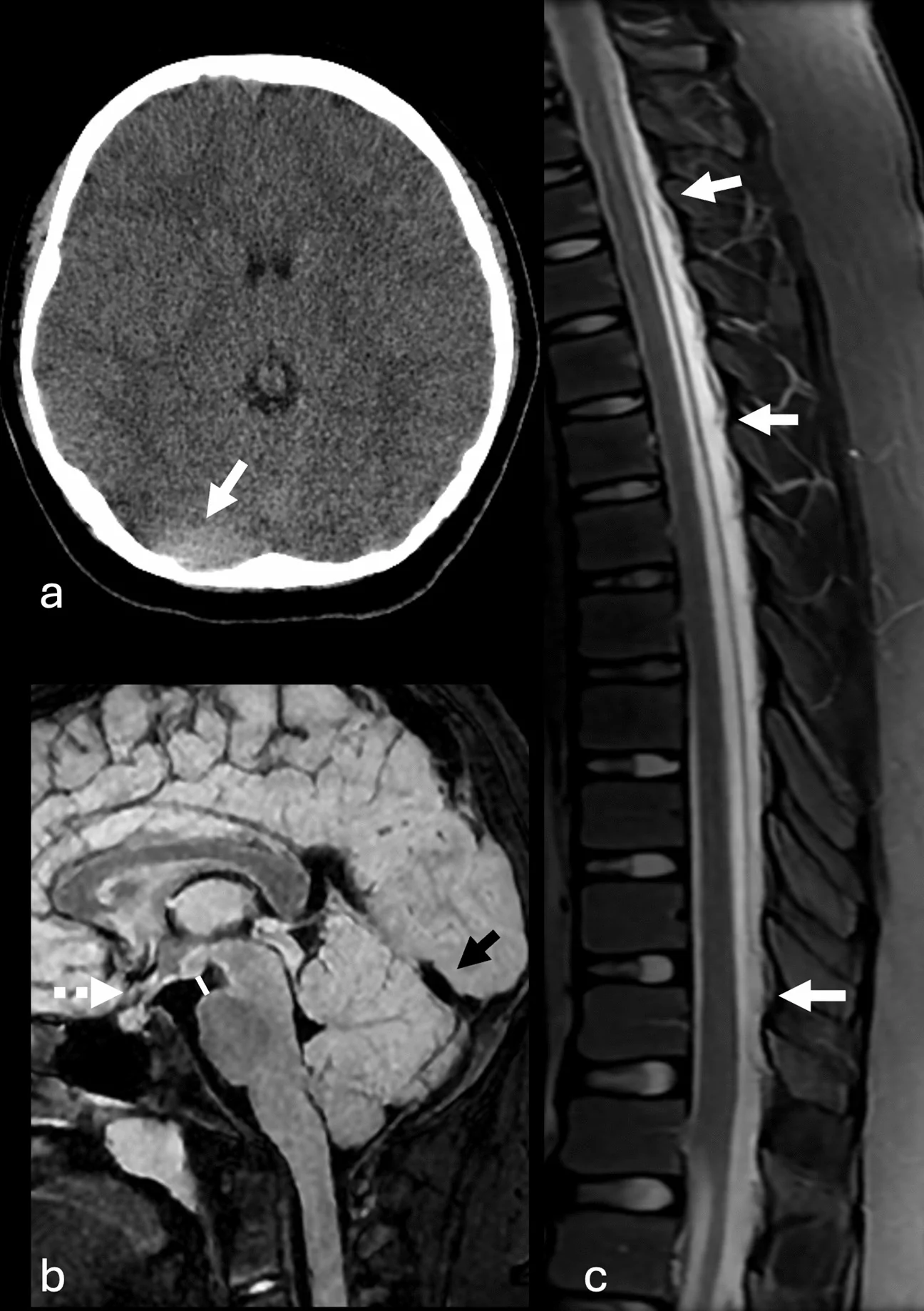
Traumatic spinal CSF leak in an 11-year-old girl who presented with headaches and vomiting after a motor vehicle collision. **a** Axial CT head shows an enlarged and hyperdense right transverse sinus (*arrow*), which was reported as a venous sinus thrombosis. **b** Sagittal FLAIR MRI was performed and did not confirm the venous sinus thrombosis but rather showed diffusely enlarged venous sinuses (*black arrow*) as well a slightly narrowed mamillopontine distance (*white line* that measured 4.7 mm [normal >6.5 mm]) and suprasellar cistern (*dashed white arrow*). These findings were recognized while the patient was on the MRI table, and sagittal T2-weighted fat-suppressed thoracic spine MR imaging was performed that showed extensive epidural fluid (**c**, *white arrows*). This case highlights that even subtle intracranial findings can lead to the discovery of a spinal CSF leak

Nonspontaneous etiologies are either iatrogenic (lumbar puncture, epidural catheter, surgery) or traumatic. Lumbar punctures can result in dural tears and yield extradural fluid on the spine MRI. While dorsal dural tears are most commonly implicated from needle punctures, it is important to note that the dural tear can also be ventral if the needle had a through-and-through course, which may require ventral epidural patching or surgery. Arachnoid blebs, a less commonly recognized leak type, can also occur secondary to a lumbar puncture where there is focal arachnoid herniating through a dural rent. Traumatic CSF leaks may occur throughout the neuro-axis, including the sacrum, and result in a dural tear with extradural fluid on spine MRI (Fig. 1).

### Spontaneous CSF leaks

Spontaneous CSF leaks are divided into dural tears, CSF-venous fistulas, and CSF-vascular malformation fistulas. Dural tears can either be ventral or lateral, and both will show extradural fluid on spine MRI. In the acute stages of these leaks, the fluid will be unorganized but can evolve into organized fluid on spine MRI in the more chronic stage due to the presence of neo-membranes that encase the collection [4].

**Fig. 2:**
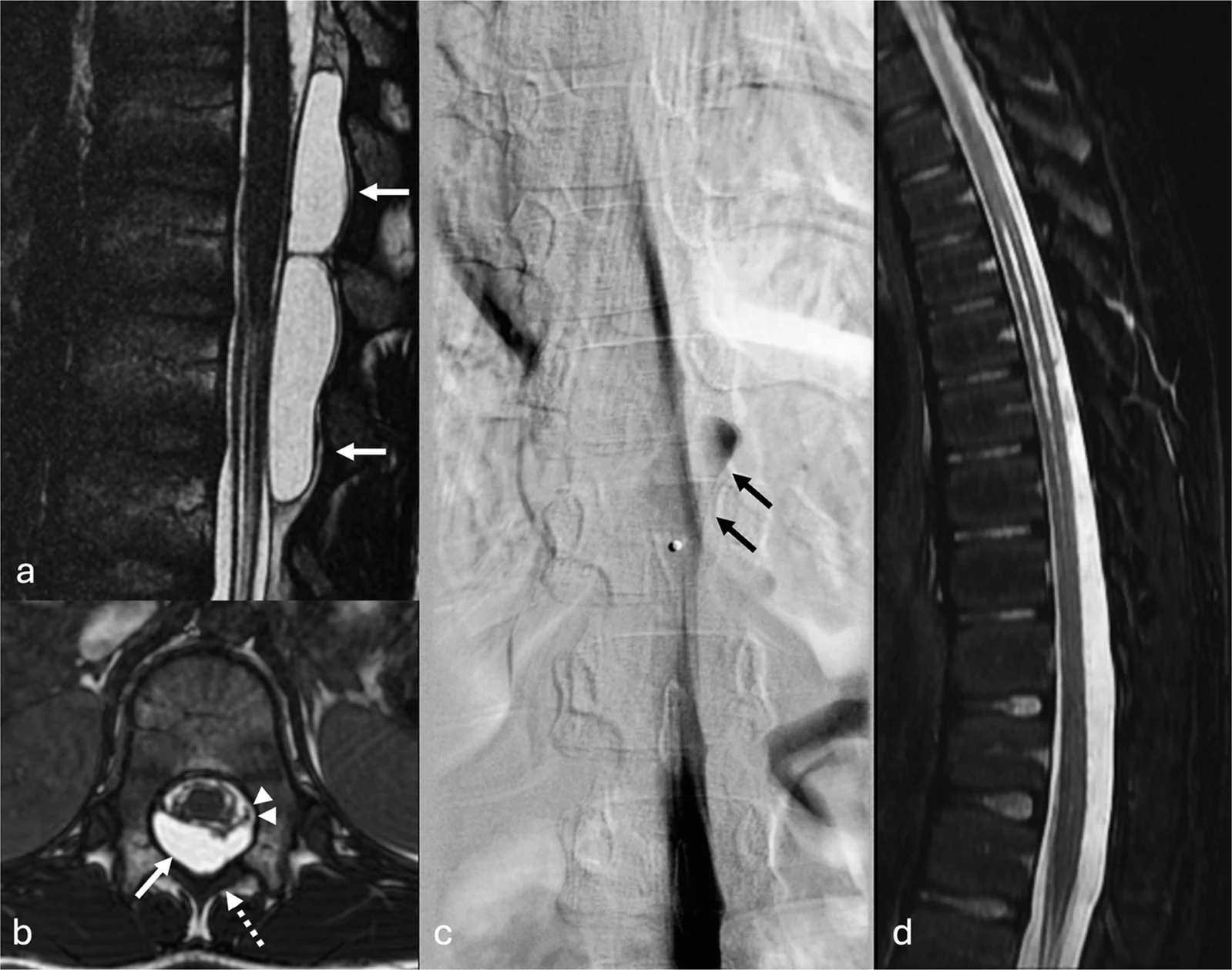
Extradural arachnoid cyst in an 8-year-old boy who had a history of enuresis and constipation. **a** Sagittal heavily T2-weighted image shows a dorsal extradural collection at the thoracolumbar junction (*white arrows*). **b** Axial heavily T2-weighted image shows the extradural collection (*white arrow*) with remodeling of the posterior elements (*dashed arrow*), a common feature of these lesions. There was suggestion of communication between the subarachnoid space and the collection at the left T12 level junction between the subarachnoid space and the extradural collection (*arrowheads*), which prompted left decubitus myelography. **c** Digital subtraction myelography (DSM) revealed a contrast jet at the left T12 nerve root level (*black arrows*) that started to fill the extradural collection more cranially. With knowledge of this leak site, the patient underwent surgery to repair the dural defect, and the postoperative sagittal T2-weighted fat-suppressed spine MRI showed resolution of the extradural collection **d**. An upper thoracic cord syrinx is also seen that was also present on the pretreatment MRIs (not shown) and may be incidental. New research has suggested that extradural arachnoid cysts may be a form of a lateral dural spinal CSF leak

Extradural arachnoid cysts are another type of extradural collection, which can result in mass effect on the cord and osseous remodeling (Fig. 2). Historically, these arachnoid cysts were designated as a separate entity from CSF leaks, but a recent study has shown these may be a form of lateral dural tear CSF leaks and are potentially better described on imaging as extradural collections as opposed to cysts [5].

CSF-venous fistulas (CVFs) are an increasingly recognized cause of spontaneous CSF leaks, particularly in adults. They represent an abnormal communication between the subarachnoid space and a spinal vein, allowing egress of CSF, and are best depicted on decubitus myelography. CSF-vascular malformation fistulas are a distinct entity where there is unregulated shunting from the spine into a venous or lymphatic malformation. These lesions are best imaged with decubitus myelography.

### Incidence and heritable disorders

Pediatric spinal CSF leaks are less common compared to adults, the latter occurring in at least 4–5 out of 100,000 people [6]. The incidence of leak types also differs. In children, dural tears are more common than CVFs [7], while in adults, both are frequently observed, and at least one study has shown that CVFs are more common [8]. Based on our clinical experience, CVFs are exceedingly uncommon in the pediatric patient. The overall rarity of spinal CSF leaks in the pediatric population and the relative dearth of scientific literature compared to that of adults is a limitation for clinical practice, and most of the discoveries and clinical applications of spinal CSF leak care are derived from the adult population.

**Fig. 3:**
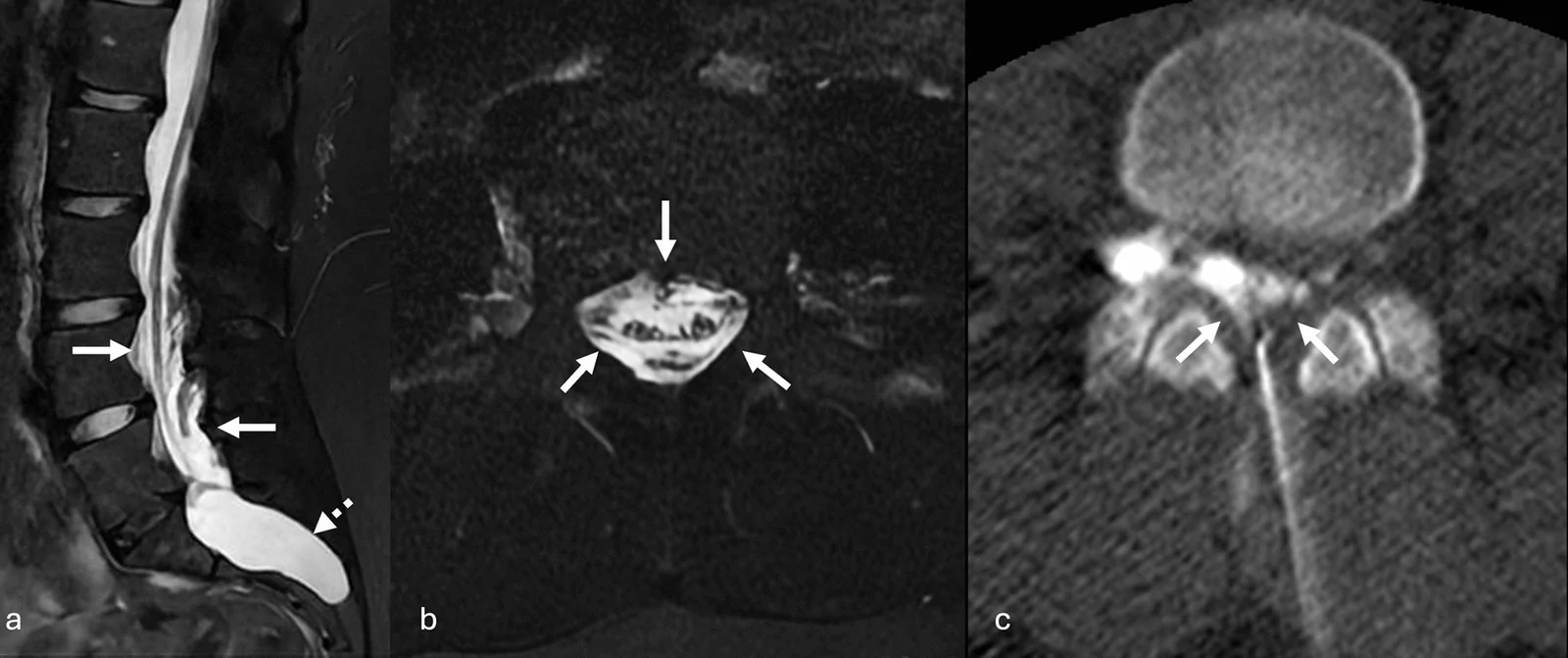
Spontaneous dural tear CSF leak in a 16-year-old boy with Marfan syndrome. **a**, **b** Sagittal and axial T2-weighted fat-suppressed images show circumferential epidural fluid in the lumbar spine (*solid arrows*) that compresses the thecal sac consistent with a CSF leak. Note the sacral dural ectasia (**b**, *dashed arrow*) that is in keeping with the connective tissue disorder and dural fragility. **c** CT-guided epidural blood patch shows a needle in the dorsal epidural space with injectate coating the epidural space (*arrows*)

Connective tissue disorders may be a risk factor for pediatric spinal CSF leaks, including Marfan syndrome, where dural fragility can occur and result in a CSF leak (Fig. 3) [9]. In patients with diffuse vascular malformations or any patient with a paraspinal vascular malformation, these could pose risk factors for the aforementioned CSF-vascular malformation fistula [10].

## Imaging protocol

### Brain MRI

Contrast brain MRI is the primary modality for intracranial evaluation of patients with suspected SIH. Although no universally accepted MRI protocol exists for SIH, there is broad agreement regarding the imaging sequences required to evaluate the characteristic intracranial findings (Table 2) [11]. Sagittal imaging is needed to assess brain sag and venous distension, and pre- and postcontrast T1-weighted imaging with gadolinium are needed to evaluate for dural enhancement. Volumetric gradient-echo techniques are highly sensitive for detecting dural enhancement but may overcall enhancement because mild physiologic dural enhancement can be more conspicuous. Spin-echo contrast sequences generally provide greater specificity. T2 or FLAIR imaging is recommended to evaluate subdural collections and alternative diagnoses [11]. Coronal T2-weighted fat-suppressed images of the orbits can help assess the optic nerve sheath fluid.

**Table 2:** MRI brain and spine imaging protocol for intracranial hypotension

Study	Recommended sequences	Purpose
Contrast brain MRI	Sagittal T1, axial T2/FLAIR, pre/postcontrast T1, coronal T2 fat-suppressed orbits	Brain sag, dural enhancement, venous distension, subdural collections, optic nerve sheaths; Bern score
Noncontrast spine MRI	3-plane STIR/fat-suppressed T2 and/or 3D heavily T2-weighted	SLEC, meningeal diverticula

### Spine MRI

The imaging protocol should include three-plane T2-weighted fat-suppressed images to increase conspicuity of the extradural fluid, and heavily T2-weighted 3D images can provide additional spatial resolution, particularly for smaller leaks such as arachnoid blebs. Noncontrast spine MRI is the mainstay of evaluation. Although contrast-enhanced imaging may demonstrate distended epidural veins, it generally provides limited additional diagnostic information.

## Imaging findings of intracranial hypotension and CSF leaks

### Brain imaging findings

**Fig. 4:**
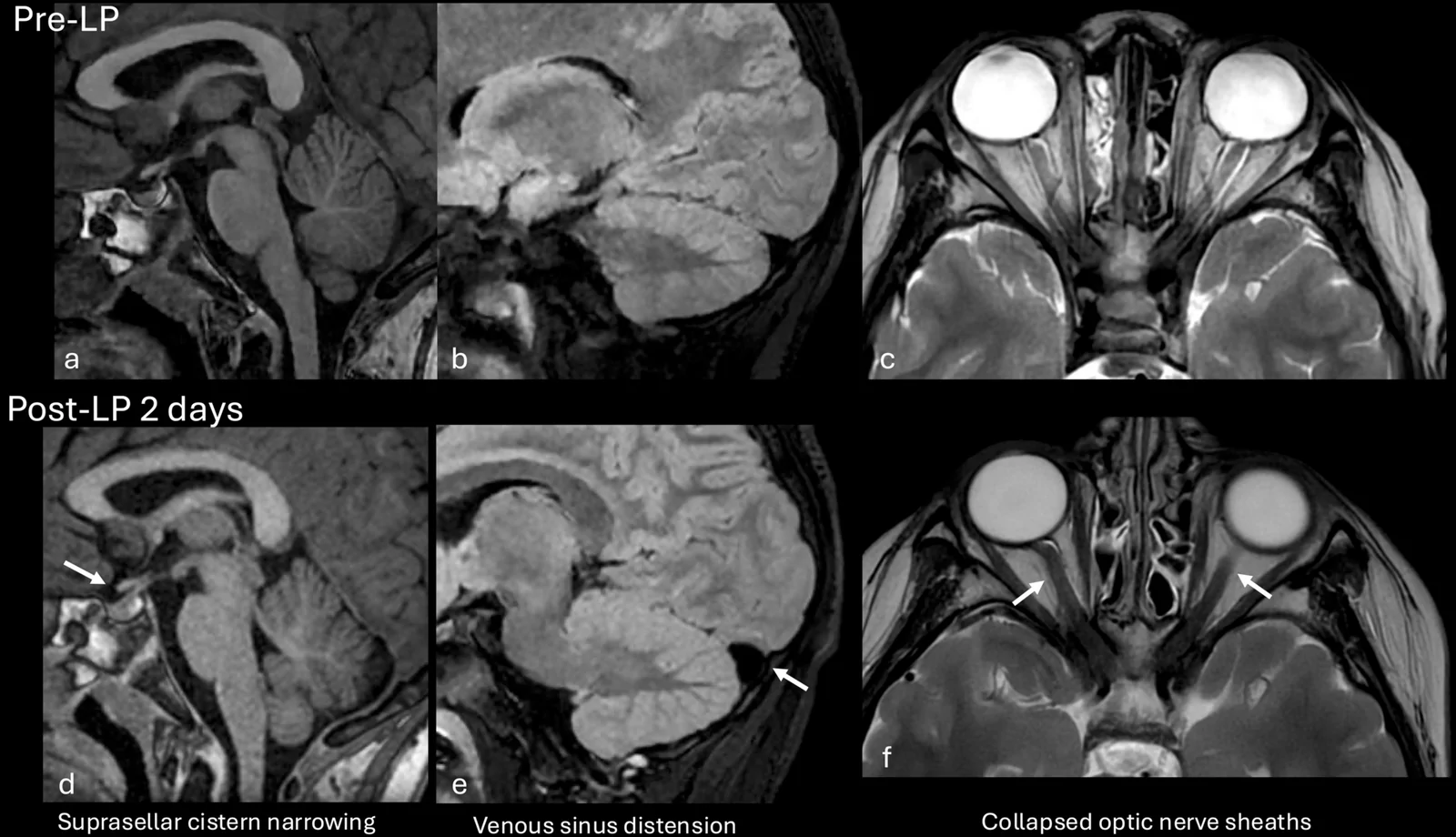
Rapid development of intracranial hypotension in a 9-year-old boy 2 days after a lumbar puncture. **a**-**c** Normal brain MRI 3 h before the lumbar puncture shows a normal suprasellar cistern distance (**a**, sagittal T1-weighted image), concave transverse venous sinus (**b**, sagittal FLAIR), and normal fluid in the optic nerve sheaths (**c**, axial T2-weighted image). **d**-**f** Postprocedural MRI performed 2 days after the lumbar puncture shows new suprasellar cistern narrowing (**d**, *arrow*, sagittal T1-weighted image), convex venous sinus distension (**e**, *arrow*, sagittal FLAIR), and collapsed optic nerve sheaths (**f**, *arrow*, axial T2-weighted image). The spine MRI (not shown) demonstrated diffuse epidural fluid throughout the total spine

The hallmark features of intracranial hypotension include brain sag, dural enhancement, venous distension, and subdural collections. An additional helpful imaging feature is the optic nerve sheath fluid, which can be collapsed in intracranial hypotension. This can be assessed qualitatively or by measuring the optic nerve sheath diameter, with one study specifying a diameter less than 4.4 mm to be a cutoff value in spontanenous intracranial hypotension (SIH) patients [12]. It is worth noting that intracranial hypotension findings can occur abruptly after the CSF leak onset (Fig. 4) and can also improve rapidly after treatment [13]. Superficial siderosis has also been described in adults with chronic SIH but appears uncommon in children.

The SIH Bern score incorporates many of these imaging findings to estimate the probability of an underlying spinal CSF leak (Table 3). There are 3 quantitative measurements (suprasellar cistern ≤4 mm [2 points], prepontine cistern ≤5 mm [1 point], and mamillopontine distance ≤6.5 mm [1 point]) and 3 qualitative features (dural enhancement [2 points], venous sinus distension [2 points], and subdural collections [1 point]). Venous sinus distension is assessed at the middle third of the dominant transverse sinus and is characterized by a convex inferior border, rather than the normally concave contour [14]. The total score categorizes patients as having a low (0–2 points), intermediate (3–4 points), or high (≥5 points) probability of an underlying spinal CSF leak [15]. Of note, the Bern score was studied and validated in the adult population, and further research is needed to determine its utility in the pediatric population.

**Table 3:** SIH Bern score brain MRI assessment

Criteria	Imaging feature	Points
Major	Venous distension	2
	Dural enhancement	2
	Suprasellar cistern ≤4 mm	2
Minor	Subdural fluid collection	1
	Prepontine cistern ≤5 mm	1
	Mamillopontine distance ≤6.5 mm	1

Probability of spinal CSF leak: low (0–2 total points); intermediate (3–4 points), high (≥5 points)

### Spine imaging findings

The main assessment for nonspontaneous and spontaneous CSF leaks on the spine MRI is to determine the presence or absence of a spinal longitudinal extradural collection (SLEC). A SLEC appears as T2-hyperintense fluid within the epidural space and may extend over multiple spinal levels. Recognition can be facilitated by identifying displacement or replacement of the normally T2-hypointense epidural fat, which is why fat-suppressed imaging is paramount. A thin dural line separates the epidural collection from the intrathecal CSF. CSF pulsations can occasionally mimic a SLEC, but these are in the subarachnoid space. Acute CSF leaks often manifest as unorganized fluid in the ventral and dorsal epidural spaces and can extend to the foramina, while chronic CSF leaks are more organized and convex in morphology.

## Imaging algorithms for CSF leak detection

### Approach for iatrogenic CSF leaks

In the setting of a presumed iatrogenic spinal leak from instrumentation, imaging is not required unless the symptoms are refractory to conservative or interventional treatments. Should imaging be required, spine MR imaging can be performed either of the affected region or the entire spine. Contrast brain MRI may or may not be needed.

### Approach for spontaneous CSF leak detection

Spinal CSF leak diagnostic algorithms may vary slightly by institution, but there are common themes that have converged in recent years given our greater understanding of CSF leaks. MRI brain and total spine serve as the initial imaging approach in many scenarios, followed by advanced myelography [11]. Our institution’s algorithm will be described.

**Fig. 5:**
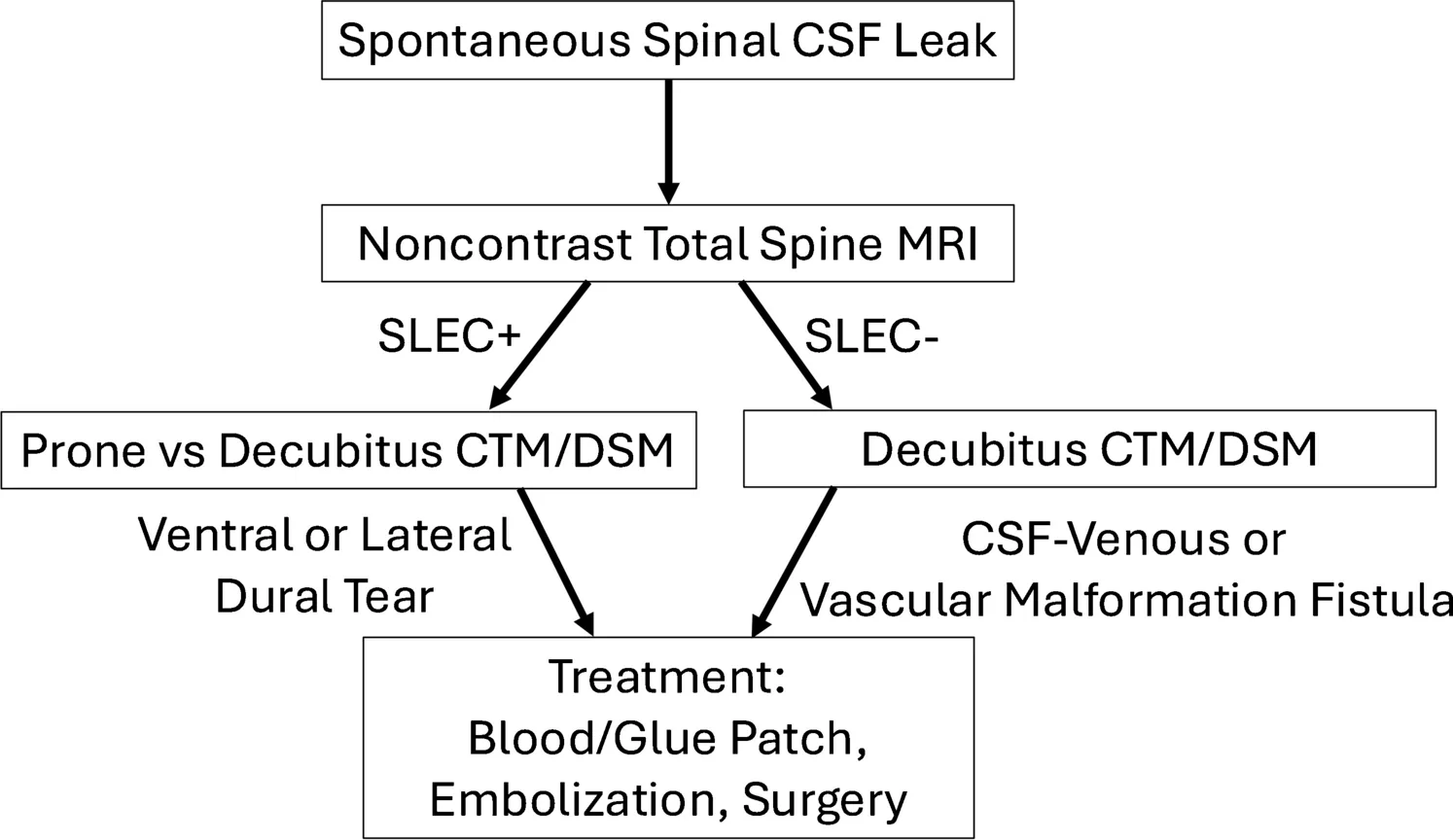
Spontaneous spinal CSF leak diagnostic and treatment pathway. When clinical findings and brain MRI raise suspicion for a spontaneous spinal CSF leak, a noncontrast total spine MRI with T2-fat suppression is performed. If there is a spinal longitudinal extradural collection (SLEC), this points to a dural tear CSF leak, and the patient is placed prone or decubitus for CT myelography (CTM) or DSM, depending on if the concern is for a ventral or lateral CSF leak, respectively. Blood/fibrin glue patching or surgery are performed. If the spine MRI does not show a SLEC, the patient is positioned decubitus for myelography to identify a CSF-venous fistula or CSF-vascular malformation fistula, followed by patching, embolization, or surgery for treatment

The diagnostic pathway of spontaneous CSF leaks is predicated on the noncontrast spine MRI (Fig. 5). If the spine MRI shows a SLEC, then the leak is often from a ventral or lateral dural tear. If the spine MRI shows no SLEC, one should scrutinize for enlarged meningeal diverticula or paraspinal vascular malformations, which may harbor an underlying CVF or CSF-vascular malformation fistula, respectively, on myelography [11].

### Myelography

**Fig. 6:**
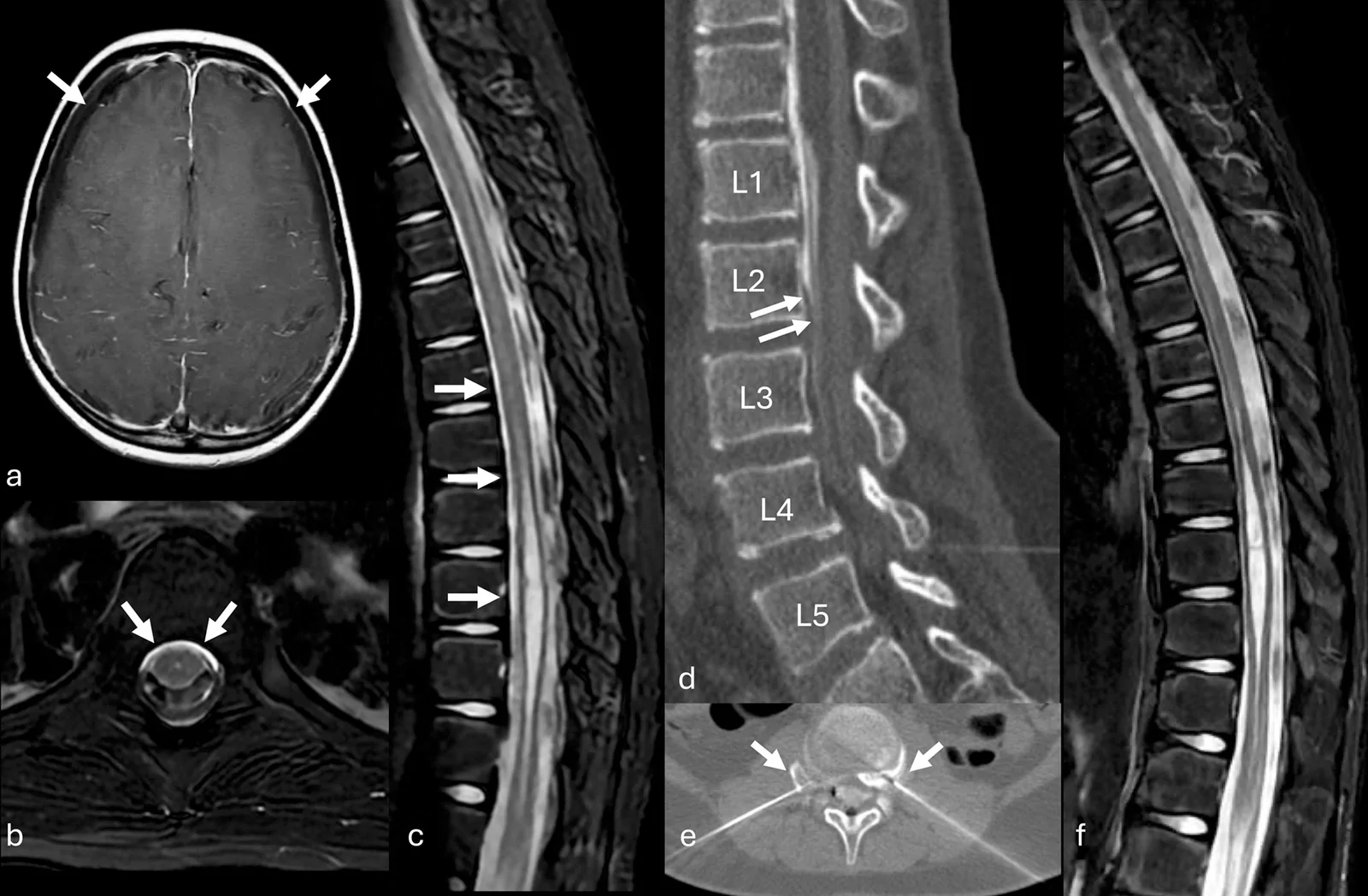
Iatrogenic spinal CSF leak in a 12-year-old girl with treated medulloblastoma who endorsed 9 months of headaches after a lumbar puncture. **a** Axial contrast-enhanced brain MRI shows bilateral subdural collections and dural enhancement (*arrows*). **b**,** c** Axial and sagittal T2-weighted fat-suppressed thoracic spine MRIs demonstrate a ventral epidural collection (*arrows*) that is partially organized, in keeping with a chronic CSF leak. A spinal cord syrinx is noted and is unrelated. **d** Prone Trendelenburg CTM with a lumbar puncture at the L4-L5 level shows a split contrast column at the L2-L3 level (*arrows*) between the subarachnoid and ventral epidural spaces consistent with a ventral CSF leak from a through-and-through lumbar puncture. The patient positioning and immediate imaging after the contrast injection were pivotal in establishing this diagnosis. In the same session, bilateral ventral epidural blood patching was performed via a foraminal approach, and injectate can be observed within the ventral epidural spaces bilaterally (**e**, *arrows*, axial CT). **f** Posttreatment sagittal T2-weighted fat-suppressed thoracic spine MRI shows resolution of the ventral epidural collection consistent with cure of the spinal CSF leak

Myelography for CSF leaks can either be performed with CT, digital subtraction, or conventional fluoroscopy. Due to the need for high spatial and temporal resolution, many centers perform CT myelography (CTM) or digital subtraction myelography (DSM). Conventional, or static, myelography, which is defined as injecting contrast under fluoroscopy, transporting the patient to CT in a delayed fashion, and scanning in the supine position, is unlikely to precisely identify a CSF leak and is not suggested. Instead, the aforementioned spine MRI is pivotal in directing the advanced myelographic approach. If there is concern for a ventral dural tear on the spine MRI, the patient is placed in a prone Trendelenburg position with the use of a foam wedge. A lumbar puncture is performed commonly at L3-L4 or L2-L3 with a 22- or 25-gauge noncutting pencil point needle, and a small volume (1–2 mL) of preservative-free iohexol contrast (Omnipaque 240; GE Healthcare) is administered, followed by 0.625 mm slice thickness images on CTM of the SLEC. A ventral dural tear is illustrated by a split contrast column extending from the subarachnoid space to the ventral epidural space. Additional contrast in 1–2 mL boluses can be administered with more focused imaging of the area of interest in a modified dynamic technique [16]. In the setting of an iatrogenic leak from a dural puncture, myelography is not required but may be needed if the CSF leak is refractory to conservative measures or epidural patching, as the leak may be at the ventral dura (Fig. 6).

**Fig. 7:**
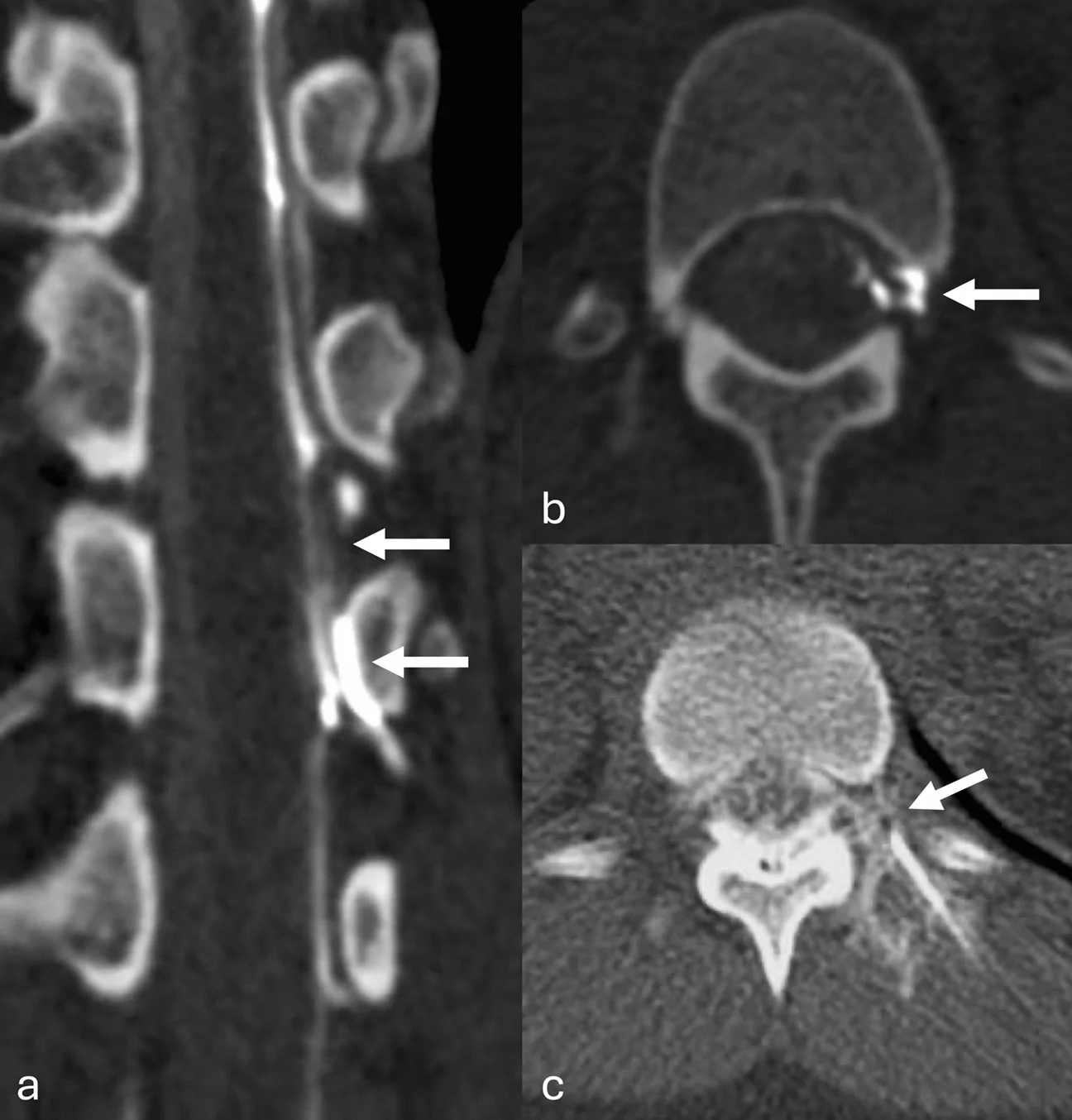
Spontaneous lateral dural CSF leak in a 25-year-old woman with left dorsolateral fluid on spine MRI (not shown). **a**, **b** Left decubitus CTM in the coronal and axial planes show a contrast jet at the left T12-L1 level (*arrows*) that extends cranially consistent with a lateral dural tear CSF leak. **c** Targeted blood patch at the left T12-L1 neural foramen (*arrow*, axial CT) and dorsal epidural space (not shown) was performed in the same session as the myelogram. The patient’s symptoms completely resolved and posttreatment brain and spine MRI (not shown) were normal

**Fig. 8:**
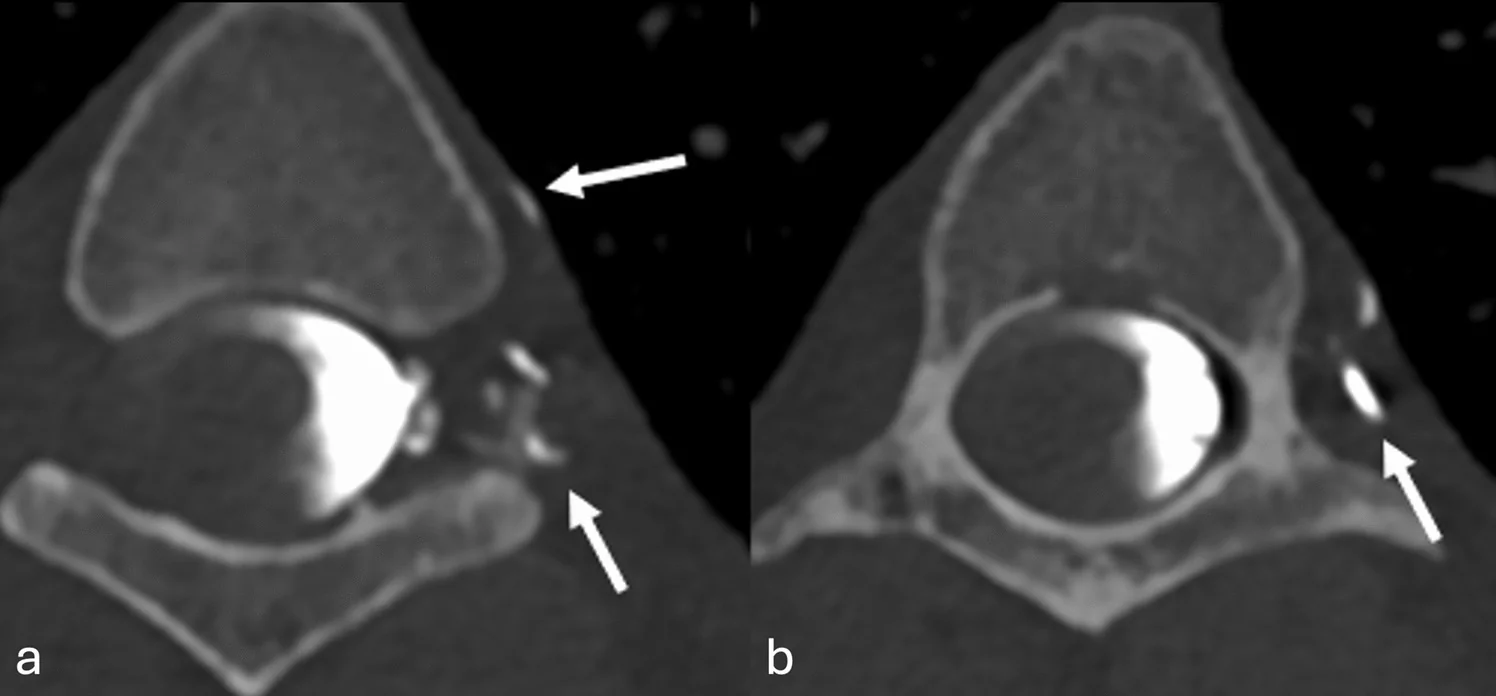
CSF-venous fistula in a 17-year-old girl with orthostatic headaches and tinnitus. **a**, **b** Left decubitus CTM images show left foraminal and segmental veins (*arrows*) consistent with a CSF-venous fistula. (Images courtesy of Ajay Madhavan, MD)

If there is suspicion for a lateral dural tear, a similar technique is performed while placing the patient in a decubitus Trendelenburg position ipsilateral to the suspected side of leak (Fig. 7). Decubitus CTM is also the mainstay for CVF evaluation (Fig. 8), and 5–8 mL of contrast are administered with imaging of the entire spine [17]. If no CVF is identified, the patient can be rotated to the contralateral side with the needle in place, and additional contrast can be injected, followed by spinal imaging [18].

Prior to myelography, contraindications to lumbar puncture and intrathecal contrast administration should be reviewed, including active infection, uncorrected coagulopathy, and elevated intracranial pressure due to a mass. Patients should also be screened for a history of seizures and medications that lower the seizure threshold in accordance with institutional guidelines. Preservative-free, nonionic, low-osmolality iodinated contrast is recommended for intrathecal administration, with contrast volume individualized according to patient size and the specific myelographic technique.

## Pediatric-specific considerations to imaging

### Contrast guidelines

There are reported contrast constraints for pediatric myelography, with at least one vendor recommending a dose range from 0.36–2.7 g of iodine, which varies by age [19]. While these data are not well validated in routine clinical practice, adherence to the recommended limits is prudent. At our institution, iohexol 240 iodine/mL is used for all pediatric CT myelograms. When a larger injectate volume is desired, the contrast is diluted with preservative-free sterile saline. Because saline does not contribute iodine, the total iodine dose is determined solely by the volume of iohexol administered (0.24 g iodine/mL), regardless of the final diluted volume, and should remain below the recommended age-specific limits.

### Ionizing radiation

Ionizing radiation is a concern when performing myelography, particularly in pediatric patients, as the entire spine may need to be imaged in certain scenarios. For dural tear evaluation, scanning can be concentrated over the SLEC levels to minimize dose. Moreover, if the leak is identified but additional imaging is needed for confirmation, the scan area can be reduced to a few spinal levels. In adult patients, some centers perform multiple sequential acquisitions of the spine to identify the leak site, which should be avoided in children. Depending on technique and operator experience, DSM may provide lower radiation exposure than CTM while maintaining high diagnostic performance [20].

### Sedation

General anesthesia or conscious sedation may be required for myelography or MR imaging. As myelography may be performed in the prone or decubitus Trendelenburg positions, communication with the anesthesia providers is needed for safe positioning of anesthesia and breathing equipment.

### Potential confusion with Chiari 1 deformity

**Fig. 9:**
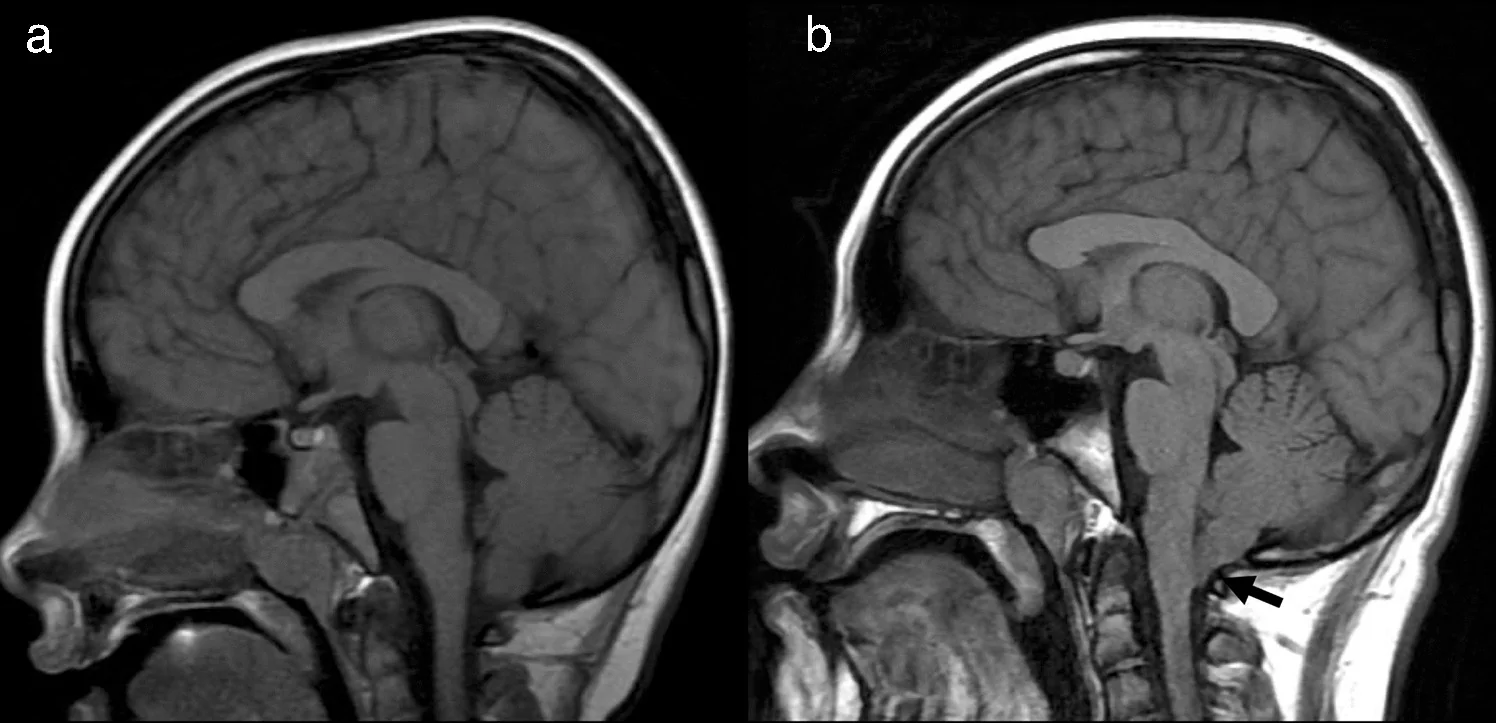
Chiari 1 deformity that developed during childhood and mimicked SIH. **a** Sagittal T1-weighted brain MRI at age 7 is normal. **b** Sagittal T1-weighted brain MRI at age 12 shows new cerebellar tonsillar descent (*black arrow)* consistent with a Chiari 1 deformity. Note that the mamillopontine distance is normal, which is preserved with Chiari 1 deformity and is often narrowed in SIH

SIH is commonly confused with Chiari I deformity in pediatric patients, particularly because the former is less common, and both can result in cerebellar tonsillar descent. Furthermore, because cerebellar tonsillar descent in Chiari 1 deformity can develop as the child grows rather than being present at birth, it may further mimic SIH (Fig. 9) [21]. The mamillopontine distance is a helpful distinguishing feature, as it is typically reduced in SIH and preserved in Chiari I deformity [22]. Moreover, there are often additional coexisting imaging findings of SIH that can help distinguish between these two entities. Another potential overlap between SIH and Chiari 1 is that both can result in the formation of a syrinx. Although the mechanism of syrinx formation in SIH remains uncertain, altered CSF flow at the craniocervical junction secondary to brain sag has been proposed [23].

## Treatment

Treatment of spinal CSF leaks includes epidural patching, transvenous embolization, and surgery. Certain types of CSF leaks may require one or more of these treatments.

### Epidural patching for dural tears

Epidural patching can be effective for nonspontaneous and spontaneous dural tears and can be performed at the affected spinal level without image guidance or with fluoroscopic or CT guidance. The decision is often based on the operator’s training as well as the spinal location for patching. Patching with CT guidance provides precise needle positioning in the epidural space and will be described. The patient is placed prone on the CT table, and grid markers are placed on the patient’s back to help with needle localization. Low-dose intermittent CT acquisitions limited to the target spinal level are typically sufficient for needle guidance while minimizing radiation exposure. After local anesthetic is administered, a 20- or 22-gauge needle is advanced to the dorsal epidural space while intermittently scanning. CT images are used to confirm that the needle tip remains within the dorsal epidural fat and outside the thecal sac. A small volume of air or diluted contrast can then be injected to confirm epidural positioning, demonstrating characteristic epidural spread without intrathecal or intravascular opacification. During blood patch administration, intermittent CT imaging is used to confirm that the injectate (mixed with contrast) remains confined to the epidural space, extends toward the site of the dural defect, and does not produce excessive compression of the thecal sac (Fig. 3). It is important to note that if the patient is under general anesthesia, there is no biofeedback from the patient, and compression of the thecal sac should be monitored and halted if significant [24].

If there is a ventral dural tear, needles can be advanced to the ventral epidural space via a foraminal approach (Fig. 6). The upper third of the neural foramen contains vasculature and nerves, and access through the lower third is advised, when possible, to avoid injury [25]. Lateral dural tears can also be treated with targeted patching via a foraminal approach (Fig. 7).

Although 20 mL has historically been recommended for lumbar epidural blood patching after post-dural puncture headache [26], this volume derives largely from obstetric/anesthesia literature and should not be interpreted as a fixed requirement for all spinal CSF leaks. The procedural goal is adequate spread of blood to the dural defect, with volume individualized according to leak location, patient size, epidural capacity, imaging appearance, and patient tolerance [24]. Fibrin glue patching, which is an off-label use, can also be administered instead of blood, or in conjunction, for any dural tear type. In general, autologous blood distributes more readily throughout the epidural space, whereas fibrin glue exhibits greater adhesive properties and may provide a more robust seal.

### Fibrin glue patching and transvenous embolization for CVFs and CSF-vascular malformation fistulas

**Fig. 10:**
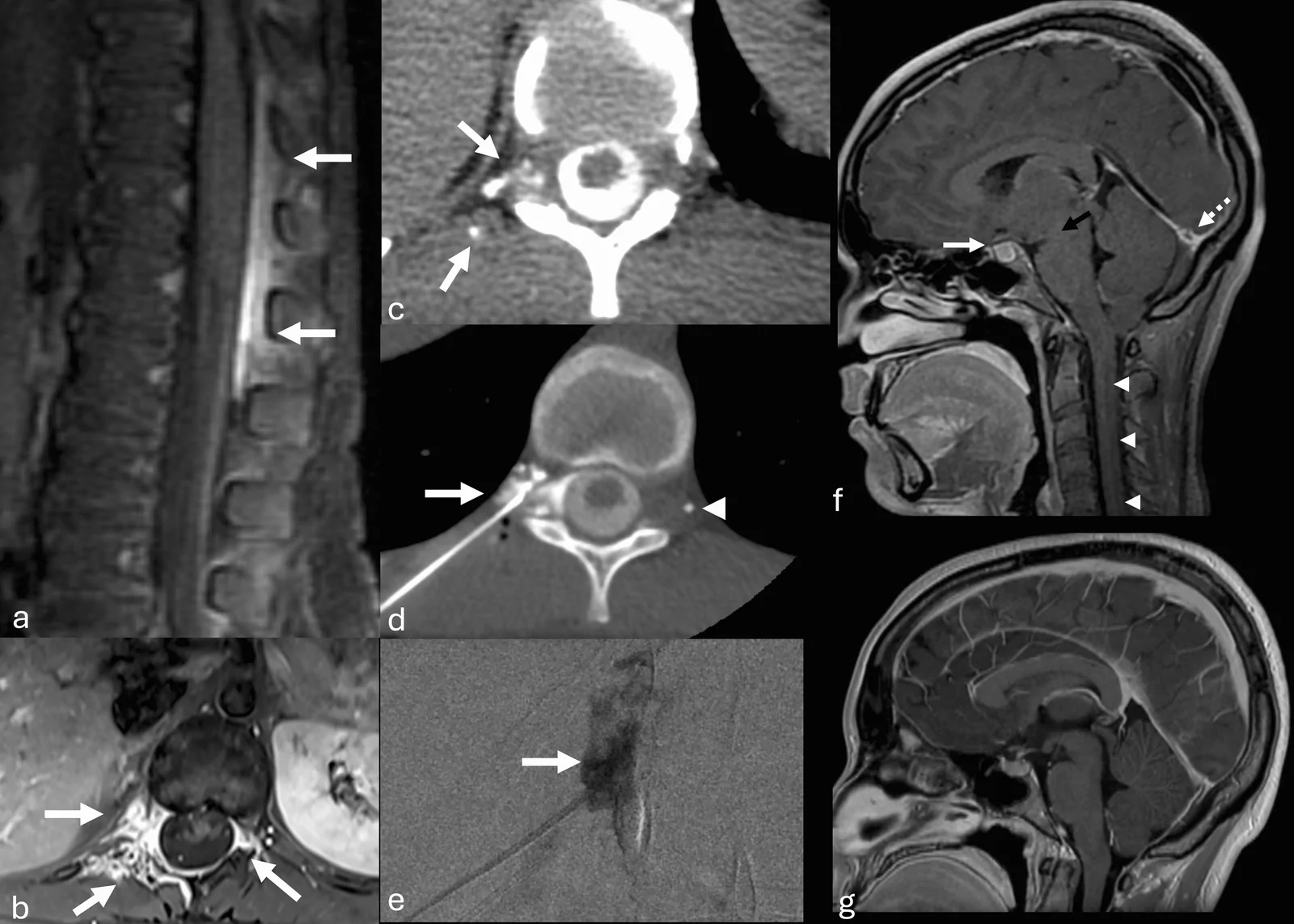
CSF-venous malformation fistula that developed between childhood and early adulthood. **a** Sagittal contrast-enhanced fat-suppressed T1-weighted spine MRI shows dorsal epidural enhancement at the thoracolumbar junction (*arrows*) in a 16-month-old girl who abruptly stopped walking. This lesion was reported as an epidural hematoma or hemangioma. The patient’s condition improved until 22 years later when she presented with headaches and was diagnosed with a “Chiari” and was referred to neurosurgery for surgical decompression. **b** Axial contrast-enhanced fat-suppressed T1-weighted spine MRI at age 23 shows circumferential epidural enhancement and dural ectasia at the T12 level extending into the right paraspinal soft tissues (*arrows*). A CSF-venous malformation fistula was suspected, and a right decubitus CTM was performed that confirmed the fistula, showing myelographic contrast in the right T12 neural foramen and paraspinal soft tissues (**c**, *arrows*). In the same session, a targeted fibrin glue patch was injected at the fistulous connection (**d**, *arrow*, axial CT). Sclerotherapy within the venous malformation (**e**, *arrow*) was performed at a later date, which resulted in complete clinical improvement of symptoms. **f** Pretreatment sagittal T1-weighted contrast-enhanced brain MRI shows narrowing of the suprasellar cistern (*white arrow*) and mamillopontine distance (*black arrow*) as well as venous sinus distension (*white dashed arrow*) and a cervical cord syrinx (*white arrowheads*). These findings resolved on posttreatment sagittal T1-weighted contrast-enhanced brain MRI **g**

As previously mentioned, CVFs are uncommon in the pediatric population. Should they occur, fibrin glue patching or transvenous embolization can be performed. Fibrin glue patching is targeted at the CVF either with an intra-cystic approach of the involved meningeal diverticulum/perineural cyst or placed at the cyst-vein junction [24, 27, 28]. Nontargeted patching or patching in the dorsal epidural space are not curative treatments for CVFs. Transvenous embolization is another technique that is commonly used for CVFs where the involved veins are selectively catheterized, and an embolic material is injected [29]. To date, there are no direct comparisons between the targeted fibrin glue patching and transvenous embolization. Both techniques are also possible for CSF-vascular malformation fistulas (Fig. 10).

### Surgery

Surgery can be performed for any of the leak types and may be necessary if the leak is refractory to the aforementioned treatments. Ventral dural tears with organized SLECs and arachnoid blebs are difficult to treat with epidural patching and commonly require surgery.

## Evaluation of patients with negative MR imaging

Patients may present with clinical symptoms suggestive of a spinal CSF leak even when MRI findings are initially negative. In this scenario, the imaging should be carefully re-evaluated, as subtle findings (e.g., venous distension) may be overlooked and are important for establishing the diagnosis and guiding subsequent management. If the MRIs are indeed normal, and there is a low-intermediate suspicion for a CSF leak, a patient discussion can be made whether to pursue myelography or empiric epidural patching, after discussing the benefits and risks (radiation exposure, anesthesia, potential dural puncture). In the adult population, there is a small subset of patients that can have definitive CVFs in the setting of a normal brain and no meningeal diverticula on spine MRI, but these are the exception. In the pediatric population, where CVFs are uncommon, most patients with a CSF leak will have either an abnormal brain MRI, abnormal spine MRI, or both [7]. This distinction should help direct the patient’s care plan, and unnecessary procedures should be minimized.

## Conclusions

Although pediatric CSF leaks are rare, recognition of their imaging manifestations is critical, as delayed diagnosis remains common. Brain and spine MRI serve as the cornerstone for evaluation and guide selection of the optimal myelographic technique for precise leak localization. Understanding the diverse spectrum of pediatric CSF leaks—dural tears, CSF-venous fistulas, and CSF-vascular malformation fistulas—is essential for accurate diagnosis and treatment planning. With modern advances in myelography, image-guided patching, and endovascular therapies, the neuroradiologist increasingly plays a central role in both the diagnosis and treatment of these patients.

## Data Availability

No datasets were generated or analysed during the current study.
